# An Innovative Approach to Enhanced Care Management for High-Need Pediatric Medicaid Members: Retrospective Cohort Study

**DOI:** 10.2196/88204

**Published:** 2026-04-22

**Authors:** Jessie L Juusola, Shefali Kumar, Meghana S Iragavarapu, Luke Mueller, Neil Batlivala, Michael K Ong, Andrey Ostrovsky, Nathan Favini

**Affiliations:** 1 Anchor Outcomes LLC, 1933 California Street, San Francisco, CA, 94109, United States; 2 Pair Team, 1459 18th Street, #206, San Francisco, CA, 94107, United States, 1 4153206716; 3 School of Computer Science, Carnegie Mellon University, Pittsburgh, PA, United States; 4 Departments of Medicine & Health Policy and Management, University of California, Los Angeles, Los Angeles, CA, United States; 5 Social Innovation Ventures, Lewes, DE, United States; 6 School of Public Health, University of California, Berkeley, Berkeley, CA, United States

**Keywords:** ECM, complex needs, HRSN, Pair Team, CalAIM, Enhanced Care Management, health-related social needs, California Advancing and Innovating Medi-Cal

## Abstract

**Background:**

The California Advancing and Innovating Medi-Cal (CalAIM) initiative supports Enhanced Care Management (ECM) for high-need pediatric populations but published evidence of the impact of ECM in pediatric populations is lacking.

**Objective:**

We evaluated a novel multidisciplinary care model (Pair Team) for delivering ECM services, focusing on implementation and early outcomes for children and adolescents enrolled in California’s Medicaid program (Medi-Cal).

**Methods:**

We conducted a retrospective, observational cohort study of Medi-Cal-enrolled children and adolescents who enrolled in Pair Team’s program between July 2022 and November 2024. Program engagement, health care engagement, and depressive symptoms were assessed using program data, electronic health records, and prescription data.

**Results:**

The main cohort included 1294 enrollees with 12 months of follow-up data (mean age 8.9 years, 50.3% (651/1294) female, 81.8% (1058/1294) experiencing homelessness). Members averaged 2.8 interactions per month with care team members over the first 3 months and 57.1% (851/1491) were still enrolled at 12 months. In the year prior to enrollment compared to the year postenrollment, the prevalence of an asthma diagnosis increased from 7.8% to 10.0% (*P=*.005), outpatient visits increased 7% (rate ratio, RR=1.07, *P*<.001), emergency department visits decreased 9% (RR=0.91, *P=*.002), and antibiotic prescriptions increased 41% (RR=1.41, *P=*.001). For those with depressive symptoms at enrollment, mean PHQ-9 score decreased from 15.4 (SD 4.7) to 10.2 (SD 6.8) after 3 months (*P*<.001).

**Conclusions:**

An innovative ECM program successfully engaged with and retained high-need pediatric Medicaid patients. Program members had higher engagement with other health care in the year following enrollment, and depressive symptoms improved. These results highlight the potential for this model to improve outcomes for the highest-need pediatric Medicaid patients.

## Introduction

Children and adolescents with complex medical and social needs, particularly those enrolled in Medicaid, face profound challenges accessing consistent, coordinated care [1]. These pediatric populations often experience fragmented health care, unmet behavioral needs, and health-related social needs such as housing instability, food insecurity, or caregiver challenges [2]. These barriers contribute to suboptimal primary and preventive care, high emergency department (ED) use, and adverse long-term outcomes [3,4]. Emerging evidence suggests that such barriers may also lead to underdiagnosis of common conditions; for example, a national analysis of Medicaid claims data found that in 17 states, asthma diagnosis rates among Medicaid-enrolled children were lower than self-reported prevalence estimates for the general pediatric population, highlighting potential missed or delayed diagnoses in this group [5].

In response to these challenges, among others for patients of all ages, California launched the California Advancing and Innovating Medi-Cal (CalAIM) initiative in 2022 for enrollees in California’s Medicaid program, Medi-Cal. A cornerstone of CalAIM is the Enhanced Care Management (ECM) benefit, which provides intensive, whole-person care coordination for Medi-Cal beneficiaries with the most complex needs [6]. Eligibility criteria extends to children and adolescents, particularly those experiencing homelessness, serious mental illness (SMI), or at risk of avoidable hospital or ED utilization [7]. Uptake among children remains low; for example, only 4040 of an estimated 95,000 eligible children in California Children’s Services (CCS) were enrolled in ECM as of 2024 [8]. Implementers also report persistent challenges with referrals, outreach, and limited infrastructure to support scalable care coordination [9]. Further, despite the inclusion of pediatric populations, limited data exist on ECM implementation and outcomes in children and adolescents.

Shortly after CalAIM launched, Pair Team, a California-based medical group and ECM provider, introduced a novel multidisciplinary care model to deliver ECM services at scale [10]. The Pair Model includes two features not required by California’s ECM program, both designed to address limitations in prior programs for high-need, high-cost patients. It supplements limited primary care access with telemedicine-based nurse practitioners (NPs) who support chronic disease management and care transitions, and it partners with community-based organizations (CBOs), providing training, tools, and funding to help address patients’ social needs like housing, food, and transportation. This model leverages care managers from local communities, along with behavioral health clinicians, NPs, and a tech-enabled platform to coordinate services. Early evaluations have shown promising outcomes among adult participants, including increased engagement with health care and improved mental health [11]. However, children and adolescents’ distinct development stages and health care utilization patterns necessitate separate evaluation of ECM’s impact in younger populations.

Accordingly, in this study, we evaluate the implementation and early outcomes of Pair Team’s ECM program for children and adolescent members (<18 y) enrolled in Medi-Cal. We assess both program engagement metrics and changes in trends in health care engagement pre- and postenrollment, overall and among subgroups with asthma and depressive symptoms, with the goal of demonstrating that a multidisciplinary, scalable approach to ECM can successfully engage pediatric populations. Our findings add to the evidence base on community-based care management for pediatric Medicaid populations and offer practical insights into adapting ECM to better serve youth with complex needs.

## Methods

### Study Design and Population

In this retrospective, observational cohort study, we analyzed metrics on program engagement, patient engagement with health care, and behavioral health outcomes for children and adolescent patients who enrolled with Pair Team. In order to enroll with Pair Team’s ECM program, patients had to be Medi-Cal members who fell within at least one Population of Focus defined by the State of California: experiencing homelessness, at risk for avoidable hospital or ED utilization (with at least three ED visits and/or two inpatient stays over 12 mo), having SMI or substance use disorder, transitioning from a youth correctional facility, enrolled in CCS or CCS Whole Child Model with additional needs beyond the CCS condition, involved in child welfare, or having intellectual or developmental disabilities [7]. Patients or their families or guardians were referred to Pair Team by local CBO partners, health system partners, or Medicaid managed care plans, and Pair Team community engagement specialists contacted them with the option to enroll in Pair Team.

To be included in the current analysis, program members must have enrolled in the Pair Team ECM program between July 1, 2022, and November 30, 2024, been under 18 years of age when they enrolled, and have been continuously enrolled in the program for at least 105 days from their enrollment date. The 105-day threshold was chosen as a proxy for a 3-month timeframe because Pair care team members flag members for disenrollment if they have not responded to outreach in the first 90 days of enrollment and then make final attempts to engage before disenrolling the patient. For analyses that included data from one-year prior to and one-year postenrollment, only program members who enrolled prior to March 15, 2024, were included, to allow for at least one year of postenrollment data to be available from a Health Information Exchange (HIE) on the date of data extraction, accounting for a 6-week buffer period.

Subgroups of members with asthma at enrollment and those with depressive symptoms were also constructed, as these are among the most prevalent chronic conditions in pediatric populations, and disproportionately so for Medicaid enrollees [12-14]. Patients who had at least one provider diagnosis of asthma (an *ICD-10* code of J45.xx) and at least one prescription for a short-acting beta 2-agonist (SABA) “rescue” medication sent to a pharmacy in the year prior to enrollment were classified as having asthma at enrollment [15,16]. Depressive symptoms were defined as having a PHQ-9 (Patient Health Questionnaire-9) score >9 at enrollment, and only those at least 12 years old were included.

### Program Description

The Pair Model takes an integrated approach to providing care to patients with complex medical and social needs. After patients are referred to the program, they begin with completing a comprehensive health and social needs assessment. They are then assigned to a care team, consisting of three bilingual Lead Care Managers (LCMs), an NP, a registered nurse (RN), and a Behavioral Health Care Manager (BHCM). Similar to community health workers, the LCMs help foster deep trust-based relationships with the patients, schedule and manage appointments, coordinate with the rest of the care team and help navigate patients through the medical and social care system. The RNs provide patient education and triage urgent health needs. The BHCMs provide behavioral health interventions and referrals as needed. NPs play a notable and differentiating role in the Pair program by supplementing the limited availability of primary care in the community; this is not a standard component of ECM. They offer virtual visits, coordinate closely with patients’ primary care providers, and provide follow-up after ED visits, hospital discharges, and for chronic disease management. The majority of interactions between patients and the care team are with LCMs, as the program is designed to focus on addressing social needs while scaling the impact of NPs, RNs, and BHCMs over a broad population.

Another distinguishing feature of the Pair Model is its partnership with CBOs, including homeless services organizations, shelters, and food banks, who join Pair Team’s value-based network. They are provided with operational training and key tools to streamline care coordination and receive funding for services provided to patients. This partnership can help quickly address social needs of patients (such as housing, transportation needs, food and financial insecurity, etc). This level of partnership is not a standard component of ECM.

Additionally, the Pair Model utilizes a custom case management platform (Arc) that allows for in-depth care planning and automation of clinical operations, is connected to HIEs and Admission, Discharge, Transfer (ADT) feeds that provide access to outside health records and alerts when patients are in the ED or hospital, and supports seamless interactions between the care team and patients via text, phone, and email.

Additional information on the Pair Model has been previously reported [11].

### Measures

Self-reported demographics and participant characteristics were collected at program enrollment in Pair Team’s proprietary case management platform. Population of Focus characterizations were specific to the pediatric member rather than the parent or guardian. Program retention rates at 3, 6, and 12 months were calculated as the percent of those who had enrolled at least 3/6/12 months prior to data extraction who were continuously enrolled 3/6/12 months postenrollment.

Program engagement was measured by the number of interactions the member or their parent/guardian had with the Pair care team, including in-person, text, phone call, and email interactions. These are logged by care team members in the custom case management platform when they conclude an interaction. Only parent/guardian interactions related to their child were attributed to the pediatric member’s engagement.

Health care visit data, including outpatient, ED, and inpatient visits, were extracted for one-year prior to and one-year postenrollment from an HIE that partners with the two largest national EHR networks: CommonWell Health Alliance and Carequality. Combined, these networks include over 75,000 provider sites and over 270 million patients across the country, including significant coverage in California. Provider sites include acute care centers, ambulatory care centers, hospitals, lab systems, pharmacies, and postacute care centers. Visits were classified as outpatient, ED, or inpatient according to the class coding of the original Consolidated-Clinical Document Architecture record. These visits did not include interactions with Pair NPs.

Medication prescription data for one-year prior to and one-year postenrollment were extracted from an HIE that partners with Surescripts. The Surescripts network covers 99% of US pharmacies and over 324 million patients across the US. Prescription rates were calculated based on the number of records of medications dispensed to a patient with unique combinations of dispense date, National Drug Code, and fill type (first fill or refill). Rates of antibiotic prescriptions were analyzed for the overall population; asthma medications including SABA rescue medications, oral steroids, and long-term controllers were analyzed for the asthma subgroup; and for the subgroup with depressive symptoms, tricyclic antidepressants, selective serotonin reuptake inhibitors, serotonin-norepinephrine reuptake inhibitors, norepinephrine-dopamine reuptake inhibitors, tetracyclic antidepressants, atypical antipsychotics, and mood stabilizers were analyzed.

Depressive symptoms were measured by the PHQ-9 [17], which was collected for all patients over 12 years old at program enrollment, and attempts to collect follow-up scores were made monthly for all members with a score >9 at enrollment. Analysis of change in PHQ-9 scores at 3 months was conducted for those in the depressive symptoms subgroup who had a follow-up PHQ-9 score in the window of 75‐135 days postenrollment. When there were multiple scores reported in that window, the one closest to 90 days post-enrollment was used for analysis.

### Statistical Analysis

We present descriptive statistics to describe the demographics of the study sample, as well as program retention and engagement rates. For binary outcomes (eg, the presence or absence of a diagnosis), we used McNemar’s test to compare pre- and postperiod diagnosis rates within individuals.

To assess changes in visit rates and medication use, we calculated the rate ratio (RR) and corresponding 95% confidence interval (CI) using the Wald method. This method evaluates whether the rate of events (eg, provider visits or number of medications dispensed) per unit time differs significantly between two time periods, accounting for the count nature and distributional assumptions of the data, assuming that the natural logarithm of the RR is approximately normally distributed. The standard error (SE) of the log-transformed RR was calculated as the square root of the sum of the reciprocals of the event counts in each group. The 95% CI was then computed as the exponential of the log rate ratio ±1.96 times the SE.

Changes in depression severity were assessed using two complementary statistical approaches applied to PHQ-9 scores collected from the same individuals at two time points. First, to evaluate changes in the mean PHQ-9 score, we used a paired t-test. Second, to assess changes in the distribution of PHQ-9 scores without assuming normality, we applied the Wilcoxon Signed-Rank test.

All tests were two-sided, and significance was defined at the .05 level. Analyses were conducted using Python, Snowflake SQL, and Microsoft Excel.

### Ethical Considerations

Since the study was a secondary analysis of previously collected, deidentified data, it was deemed exempt from ethics oversight by the WCG Institutional Review Board (1-1952683-1).

## Results

### Sample Characteristics

**Figure 1.**
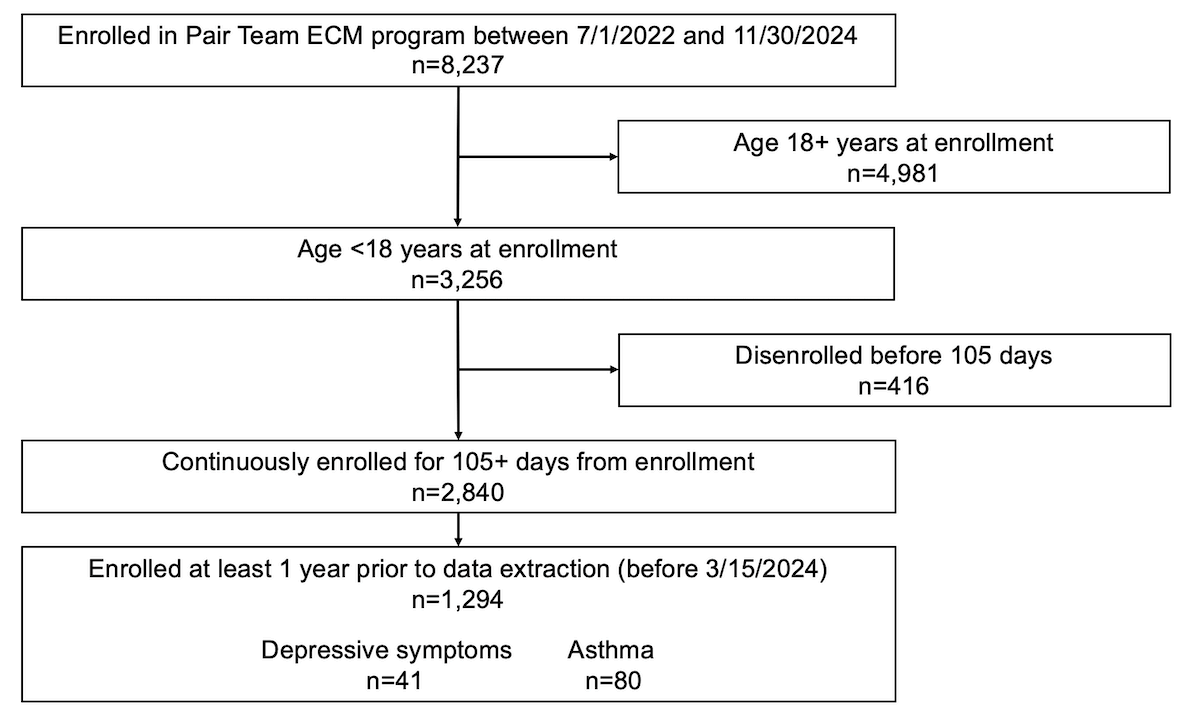
Member enrollment and study participation flow chart.

A total of 3256 patients under 18 years old enrolled in the Pair Team ECM program between July 1, 2022, and November 30, 2024. Of those, 2840 (87.2%) were still enrolled 105 days later, and 1294 had 12 months of follow-up data (Figure 1). Characteristics for the cohort of 1294 are shown in Table 1. The mean age of the study participants at program enrollment was 8.9 years (SD 5.0), with 35.2% (456/1294) being 12‐17 years old. The study population was 50.3% (651/1294) female and 46.8% (606/1294) were known to be Hispanic or Latino. The majority (81.8%, 1058/1294) were experiencing homelessness at enrollment and 25.7% (332/1294) met criteria for multiple Populations of Focus. A total of 80 (6.2%) were classified as having asthma at enrollment, and 41 had depressive symptoms at enrollment (9.0% of those 12‐17 y old, 41/456).

**Table 1.** Description of study cohort.

Characteristic	Overall (n=1294)
Sex at birth, n (%)	
Female	651 (50.3)
Age, n (%)	
0‐4	308 (23.8)
5‐11	530 (41.0)
12‐17	456 (35.2)
Age (years), mean (SD)	8.9 (5.0)
Race or Ethnic group, n (%)	
Black or African American	101 (7.8)
Hispanic or Latino	606 (46.8)
White	45 (3.5)
Other^a^	44 (3.4)
Unknown	498 (38.5)
Preferred language: Spanish, n (%)	492 (38.0)
Population of focus, n (%)^b^	
HU^c^	188 (14.5)
Homeless	1058 (81.8)
SMI^d^	269 (20.8)
Other	32 (2.5)
Multiple PoF^e^	332 (25.7)
Individuals with an asthma diagnosis, n (%)	80 (6.2)
Individuals with depressive symptoms, n (%)	41 (3.2)^f^

a “Other” Race or Ethnic Group includes American Indian or Alaskan Native, Asian, Islander, and Other.

b Participants could be part of multiple PoFs; total does not sum to 1294 or 100%.

c HU: high utilizers.

d SMI: serious mental illness.

e PoF: Population of Focus.

f Only those age 12‐17 years were eligible for this cohort; 9.0% of that age group had depressive symptoms.

### Program Engagement

As noted above, retention after 3 months of enrollment was 87.2% (2840/3256). Among the 2726 pediatric patients who enrolled in the program at least 6 months prior to data extraction, retention at 6 months was 71.3% (1943/2726). Among the 1491 who enrolled at least 12 months prior to data extraction, retention at 12 months was 57.1% (851/1491). In the first 3 months after enrollment, pediatric members or their parent/guardian engaged with the Pair care team on average 2.8 times per month. Average engagement over 6 months for those enrolled at least 6 months was 2.4 interactions per month, and there were 2.2 interactions per month in the year postenrollment for those enrolled at least 12 months. Most pediatric member or parent or guardian interactions were with LCMs (84.1%, 9436/11215 in the first 3 mo, 86.8%, 24045/27692 over 12 mo). The proportion of interactions that were with RNs or NPs or BHCMs increased over time from 2.7% (307/11215) for each in the first three months to 3.3% (925/27692) with RNs or NPs and 3.2% (888/27692) with BHCMs over 12 months. The remainder of interactions were with outreach or intake specialists or other staff.

### Engagement With Health Care

We assessed changes in level of engagement with health care by calculating changes in rates of asthma diagnoses, healthcare visit rates, and medication use between one-year prior to and one-year postenrollment.

**Figure 2.**
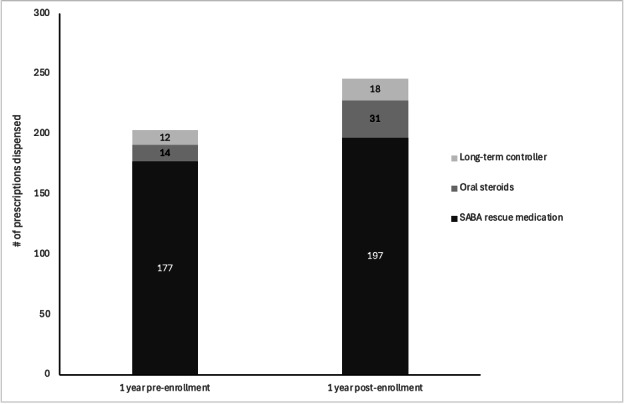
Asthma medication prescriptions by category.

The prevalence of an asthma diagnosis in the overall sample increased from 101 (7.8%) in the year prior to enrollment to 129 (10.0%) in the year post (*P*=.005). For those classified as having asthma at enrollment, prescriptions dispensed increased from 203 to 246, representing an increase of 21% (RR=1.21, 95% CI 1.01‐1.46, *P*=.01) from the pre- to postperiod. The increasing trend was seen across medication categories (SABA rescue medications, oral steroids, and long-term controllers) (Figure 2).

Health care visit rates for the overall population differed between the year prior to and the year postenrollment. Outpatient visits increased from 3898 to 4159, representing a 7% increase from the pre- to postperiod (RR=1.07, 95% CI 1.02‐1.11, *P*<.001). Conversely, acute care visits decreased. From the pre- to postperiod, ED visits decreased from 1220 to 1115, representing a decrease of 9% (RR=0.91, 95% CI 0.84‐0.99, *P*=.002). Inpatient visits were directionally lower postenrollment, decreasing from 60 to 50, representing a decrease of 17% (RR=0.83, 95% CI 0.57‐1.21, *P*=.14).

For the overall sample (n=1294), antibiotic prescriptions dispensed increased from 563 to 794, representing an increase of 41% (RR=1.41, 95% CI 1.27‐1.57, *P*=.001) from the pre- to postperiod. Amoxicillin, cephalexin, azithromycin, amoxicillin and clavulanate potassium, and erythromycin were the most common prescriptions dispensed in both the pre- and postperiods.

### Behavioral Health Outcomes

In the subgroup with depressive symptoms at enrollment (PHQ-9 >9) and a follow-up PHQ-9, average PHQ-9 score decreased from 15.4 (SD 4.7) at enrollment to 10.2 (SD 6.8) at 3 months follow-up (*P*<.001, n=38) (Table 2). At follow-up, 47.4% (18/38) of the subgroup had a PHQ-9 score categorized as none to mild depressive symptoms (PHQ-9 <10) (*P*<.001), and the percentage reporting any suicidal ideation decreased from 50.0% to 15.8% (*P*<.001). The proportion of patients reporting severe depressive symptoms in this subgroup decreased from 28.9% (11/38) at enrollment to 5.3% (2/38) at follow up (*P*<.001). We assessed change in mental health medication usage for the full cohort of 41 with depressive symptoms at enrollment. Prescriptions dispensed for this cohort directionally decreased from 72 to 52, representing a 28% decrease from the pre- to postperiod (RR=0.72, 95% CI 0.51‐1.03, *P*=.17).

**Table 2.** Pre- versus postenrollment behavioral health outcomes.

Variables	Enrollment	3-month follow-up	*P* value^a^
Patients with enrollment and follow-up score, n (%)	38 (100.0)	38 (100.0)	—^b^
PHQ-9 score, mean (SD)	15.4 (4.7)	10.2 (6.8)	<.001
Depressive symptom severity, n (%)			<.001
0‐9: None-mild	0 (0)	18 (47.4)	
10‐14: Moderate	18 (47.4)	7 (18.4)	
15‐19: Moderately severe	9 (23.7)	11 (28.9)	
20‐27: Severe	11 (28.9)	2 (5.3)	
Patients with suicidal ideation, n (%)	19 (50.0)	6 (15.8)	<.001

a For continuous variables, a paired *t*-test was used to calculate *P* values; for categorical variables, the Wilcoxon signed-rank test was used.

b Not applicable.

## Discussion

### Principal Findings

Results from this real-world study demonstrate that a multidisciplinary, scalable approach to ECM can meaningfully connect with and engage high-need pediatric Medicaid patients. The Pair Model enrolled over 3000 pediatric patients eligible for ECM in the first two years of the program, and enrollment continues to grow over time as Pair Team scales. Nearly 60% of those who enrolled were still enrolled one year later, and engagement with the care team was sustained over time, with an average of 2.2 interactions per month over the year after enrollment. These results are especially meaningful given that over 80% of those enrolled were experiencing homelessness at the time of enrollment, as populations experiencing homelessness are generally considered difficult to engage [18,19].

The differences seen in asthma diagnoses, health care visit rates, and rates of medication use in the year prior compared to the year postenrollment suggest that the Pair Model facilitates improved access to and better engagement with the healthcare system. Asthma is a condition that is more prevalent in low-income children, and diagnosis is important for proper management [13,20]. The prevalence of a record of asthma diagnosis in this study increased from 7.8% in the year prior to enrollment to 10.0% in the year postenrollment, which aligns with other estimates of asthma rates of 10% in Medicaid pediatric populations [21,22]. This suggests that asthma may have been underdiagnosed in this population prior to enrollment. It is well understood that utilizing primary and preventive care effectively leads to optimal health outcomes and lowers costs, but high ED utilization continues to be a larger problem in Medicaid populations than in commercially-insured populations [22,23]. In this study, we saw that Pair Team program members had lower ED visit rates in the year postenrollment than pre, and shifted to higher rates of outpatient visits in the post-enrollment period. This suggests that their access to and engagement with primary care improved after enrollment. While overuse of antibiotics is a problem, including in Medicaid populations, it is also important that pediatric populations have access to appropriate prescribing. The 41% increase in antibiotic prescriptions dispensed seen in this study corresponds to per-patient rates increasing from 0.4 in the year prior to enrollment to 0.6 in the year postenrollment. This brings antibiotic use in alignment with the national average of 598 prescriptions per 1000 persons for those under 18 years in 2022, and is still materially lower than prescribing rates seen in some other Medicaid populations [24,25]. However, we are unable to definitively say how much of the increase in antibiotic prescriptions is appropriate prescribing as opposed to overuse.

Adolescents experiencing complex social or medical needs, such as homelessness, are at higher risk for mental health challenges than those who are not, yet they often experience barriers to appropriate behavioral health care [19]. These barriers are multifaceted, and impacted by housing situation, access to technology, and cultural disconnection from care providers [26]. The Pair Model’s emphasis on bilingual LCMs and BHCMs seeks to address these barriers by fostering trust with patients and referring them to care providers who understand the social factors they are facing. While the sample size for those with depressive symptoms in this study is not large, the improvements in PHQ-9 results are impressive and seen in parallel with directional decreases in medication usage, suggesting that the Pair Model approach of addressing social needs may have a meaningful impact on the mental health status of Medicaid adolescents.

### Limitations

This study has several limitations, as is typical of observational studies conducted with real-world data. First, as a one-arm observational study, we did not have a control group, and as such we were unable to examine a cause-effect relationship. Changes in outcomes could thus be attributable in part to regression to the mean and confounding variables. Because of this, we focused our analysis on engagement metrics, and we plan to conduct controlled studies focusing on health outcomes in the future. Second, despite the large overall sample size, the asthma and depressive symptom cohort sizes were small, and as such findings for those cohorts should be interpreted cautiously. Additionally, PHQ-9 scores were only available for a subset of the group; however, this was mitigated by the large effect size seen between baseline and follow-up scores. Lastly, we were limited to the data that was available either from the program itself or the HIE partner. While the HIE partner has extensive coverage over provider sites, it does not include every provider site and thus may miss some provider visits. Future analyses could prospectively collect data for outcomes of interest, for example pulmonary function test results for asthma, or utilize a comprehensive medical claims database in order to capture all health care utilization.

Despite these limitations, this study meaningfully strengthens the current evidence base for ECM in high-need pediatric populations. Most of the limited literature on ECM in pediatrics involves children with medical complexity rather than more broadly-defined complex needs [27]. Research on pediatric patients with complex needs is sparse, but some initial studies of care coordination for children and adolescents with special health care needs have demonstrated promising results, just as this study has [28].

### Conclusions

In summary, this study demonstrates that a community-integrated, technology-enabled approach to ECM can successfully engage high-need pediatric Medicaid patients. Despite the inability to draw causal conclusions from this study, the study findings, in particular the high retention and engagement rates with the program, improvements in how patients engaged with health care, and improvements in depressive symptoms, provide an early indication that the Pair Model has the potential to improve outcomes for these patients. Future studies should examine additional health outcomes as well as include control groups in order to build a more robust evidence base evaluating this approach to ECM.

## Abbreviations

ADT: Admission Discharge Transfer
BHCM: Behavioral Health Care Manager
CalAIM: California Advancing and Innovating Medi-Cal
CBO: Community-Based Organization
CCS: California Children’s Services
ECM: Enhanced Care Management
ED: emergency department
HIE: Health Information Exchanges
LCM: Lead Care Manager
NP: nurse practitioner
PHQ-9: Patient Health Questionnaire-9
RN: registered nurse
RR: rate ratio
SABA: short-acting beta 2-agonist
SMI: serious mental illness
